# The Effect of Aromatic Hydrocarbon Receptor on the Phenotype of the Hepa 1c1c7 Murine Hepatoma Cells in the Absence of Dioxin

**Published:** 2007-09-18

**Authors:** Feng Wang, Ruixue Zhang, Shengli Shi, Oliver Hankinson

**Affiliations:** 1 Department of Pathology and Laboratory Medicine, Jonsson Comprehensive Cancer Center, University of California, Los Angeles, California 90095, U.S.A; 2 Molecular Biology Institute, University of California at Los Angeles

**Keywords:** albumin, aromatic hydrocarbon receptor (AhR), cell cycle, Cyp1a1, DNA microarray, morphology

## Abstract

The aromatic hydrocarbon receptor (AhR) mediates biological responses to certain exogenous ligands, such as the environmental contaminant 2,3,7,8-tetrachlorodibenzo-p-dioxin (TCDD), and has also been demonstrated to modulate the cell cycle and differentiated state of several cell lines independently of exogenous ligands. In this study, we used DNA micorarray analysis to elucidate the profile of genes responsive to the expression of unliganded AhR by re-introducing AhR into an AhR-deficient mouse derivative (c19) of the mouse hepatoma cell line Hepa1c1c7. 22 gene products were up-regulated and 8 were down-regulated two-fold or more in c19 cells infected with a retroviral vector expressing mouse AhR. Surprisingly, expression of genes involved in cell proliferation or differentiation were not affected by introduction of AhR. AhR also did not restore expression of the albumin gene in c19 cells. Introduction of AhR into c12, a similar AhR-defective mouse hepatoma cell line, also did not restore albumin expression, and furthermore, did not lead to changes in cellular morphology or cell cycle parameters. These observations fail to support the notion that unliganded AhR regulates proliferation and differentiation of liver-derived cells.

## Introduction

2, 3, 7, 8-tetrachlorodibenzo-p-dioxin (TCDD) elicits a variety of toxic, teratogenic, and carcinogenic responses in exposed animals and in humans (DeVito and Birnbaum, 1994). In certain cultured cells, TCDD shows marked effects on the regulation of cell cycle progression and terminal differentiation. It also induces thymocyte apoptosis, keratinocyte proliferation, and inhibition of estrogen-dependent proliferation in breast cancer cells (Milstone and LaVigne, 1984; Wiebel et al. 1991; Gaido et al. 1992; Safe 1995).

It is widely accepted that most, if not all effects of dioxins are mediated by a cytosolic receptor known as the aryl hydrocarbon receptor (AhR). AhR is a ligand-activated transcription factor that forms a transcriptionally active heterodimer with the aryl hydrocarbon nuclear translocator (ARNT) (Hoffman et al. 1991; Reyes, 1992). In the cytosol, the unliganded AHR is found in a complex with two HSP90 molecules, the co-chaperone protein p23, and the hepatitis B virus X-associated protein 2 (XAP2) (Meyer et al. 1998; Kazlauskas et al. 1999). Ligand binding disrupts this complex and causes nuclear translocation of the AHR. The nuclear AHR/ARNT heterodimer binds to 5′-CACGCNA/C-3′ DNA motifs (termed DREs or XREs) in the regulatory regions of the *CYP1A1, CYP1B1*, *CYP1A2* and *CYP2S1* cytochrome P450 genes, and several genes coding for phase II detoxification enzymes, such as glutathione *S*-transferase, NAD(P)H-dependent quinone oxidoreductase-1, aldehyde dehydrogenase-3, and others, and activates their transcription (Sutter and Greenlee, 1992; Hankinson, 1995; Rivera et al. 2007).

AhR has been reported to associate with retinoblastoma (RB) both *in vivo* and *in vitro*, and synergizes with RB to repress E2F-dependent transcription and to induce cell cycle arrest in a number of cell lines, although the effects of AhR on the cell cycle vary in different reports (Puga et al. 2000a; Shimba et al. 2002; Huang and Elferink, 2005). In mouse hepatoma Hepa lclc7 cells, in the absence of exogenous ligands, AhR was reported to accelerate cell proliferation, and modulate the differentiated state (Ma and Whitlock, 1996). However, the mechanisms underlying the effects of unliganded AhR on cell proliferation and differentiation of Hepa 1c1c7 cells remain to be established. In this study, we re-introduced AhR into an AhR-defective B mutant of Hepa 1c1c7 cells (Hankinson, 1983). The B mutants are defective in the expression of AhR mRNA, and have been hypothesized to be defective in the activity of a transcription factor or chromatin modification factor required for transcription of the AhR gene (Zhang et al. 1996). We then elucidated the profile of AhR-responsive genes using DNA microarray analysis. Furthermore, we studied the potential effects of AhR on the phenotype of the mouse hepatoma cells, including cell cycle, morphology and the expression of albumin.

## Materials and Methods

### Materials

The plasmids pMFG-AhR and pCMMP-lacZ were kind gifts from Drs. James Whitlock and Richard C. Mulligan, respectively (Ma et al. 1996, Klein et al. 2000).

### Cell culture

Wild-type Hepa 1c1c7 and B mutants cells (c12 and c19) were grown in alpha minimal essential medium containing 10% fetal bovine serum (FBS).

### Retrovirus preparation and infection

293T cells were grown in DMEM with 10% FBS and penicillin/streptomycin. Cells were grown to 50% confluency in 10-cm dishes and transfected with the retroviral vectors and a packaging vector pCLeco using a standard calcium-phosphate method. The medium was replaced 24 h post-transfection. The virus-containing supernatants were collected 36 and 48 h after transfection, filtered through a 0.45-μm syringe filter, and stored at 4 °C. For retroviral infection, B mutant cells (1.0 × 10^5^/well) were seeded in six-well plates, and 2 ml of virus supernatant containing 8 μg/ml of polybrene was added to the cells.

### Gene chip analysis

Total RNA was extracted from each sample with the TRIzol Reagent (Invitrogen, Carlsbad, CA, U.S.A). Two weeks after infection, RNA was extracted from infected B mutant cells. The RNA was cleaned with RNeasy Mini Kit (Qiagen Inc., Valencia, CA, U.S.A.). RNA from c19 cells infected with pMFG-AhR or pCMMP-LacZ was converted to biotinylated cRNA (Bioarray High Yield RNA Transcription Labeling Kit; Enzo Diagnostics, Farmingdale, NY, U.S.A.) according to the manufacturer’s protocols. Following fragmentation and quality confirmation with the Affymetrix Test3 Array, each biotinylated cRNA was hybridized to a Mouse Genome U74Av2 chips from Affymetrix (Santa Clara, CA), containing a total of 12,488 probe sets. Following hybridization, each chip was washed, stained with streptavidin-phycoerythrin, and scanned with a probe array scanner. Microarray expression data were generated with Affymetrix Microarray Suite 5.0 software.

### Data analysis

Microarray expression data were analyzed with DNA-Chip Analyzer (dChip) version 1.3 (Li and Wong, 2003). Gene expression results from B-mutant cells infected with pCMMP-lacZ (Basal or B) or pMFG-AhR (Experimental or E) were normalized and compared, with each group containing the data obtained from a single chip. The ‘Invariant Set Normalization’ method, which chooses a subset of probes with small rank difference in all arrays, was used to perform cross-chip normalization. Comparison was done by computing the expression fold-difference for each gene and listing those that show larger than 2.0 fold increase or decreases in activity. Genes with differences of expression levels (E-B or B-E) lower than 20 were excluded to get reliable fold-difference results. The gene categories were identified from gene ontology using NIH DAVID (http://apps1.niaid.nih.gov/david/).

### Cell cycle analysis

The cells were trypsinized and suspended in PBS. An aliquot (1 × 10^6^ cells) was placed into a centrifuge tube. After centrifugation, the cells were suspended and stained in 1 ml of hypotonic staining buffer (0.1% sodium citrate, 0.3% Triton-X 100, 0.01% propidium iodide (PI), 0.002% ribonuclease A) for 30 minutes at 4 °C, and then subjected to flow cytometry using CellQuest software on LSRII cytometer (BD Biosciences). DNA content was determined using ModFit (Verity Software House, Topsham, ME).

### Reverse transcription and real-time PCR

Total RNA was prepared from the cells using the RNeasy Micro kit (Qiagen). 1.5 μg of total RNA was reverse-transcribed using a Super script III Reverse Transcriptase kit (Invitrogen). Real-time PCR was performed using the SYBR Green Master Mix (Applied Bio-Science). The primer sets for RT-PCR are as follows: 5′-AGGTGTGATGGTGGGAATGG-3′, 5′-GCCTCGTCACCCACATAGGA-3′ for β-actin; 5′-AGATGCAGCCAGATCCGCAT -3′, 5′-GTTCTTGCCCATCAGCACC -3′ for the ribosomal 36B4 gene; 5′-AGAAGGTCACTCTCTTTGGTTTGG-3′, 5′-GCAGCAAGATGGCCAGGAA-3′ for CYP1A1; 5′-CCGTGTGACTGGCATCGATTAT-3′, 5′-CATGCCACTGCCAACTTAGGAA-3′ for albumin.

### Immunoblotting

Whole-cell lysates were prepared using lysis buffer (25 mM Hepes, PH 7.4, 1 mM EDTA, 400 mM NaCl, 1 mM DTT, and 1% Triton X-100) and resolved on 8% SDS polyacrylamide gel (50 μg/lane). The AhR protein was detected in whole-cell extracts using a 1:1000 dilution of an immunoaffinity purified rabbit polyclonal AhR antibody made in our laboratory.

## Results

### DNA microarray analysis of AhR-dependent gene expression

**Figure 1:**
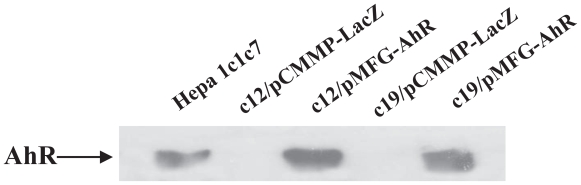
Immunoblot for AhR. Proteins were extracted from whole cells and subjected to immunoblot analysis using an anti-AhR antibody prepared in our laboratory.

**Figure 2:**
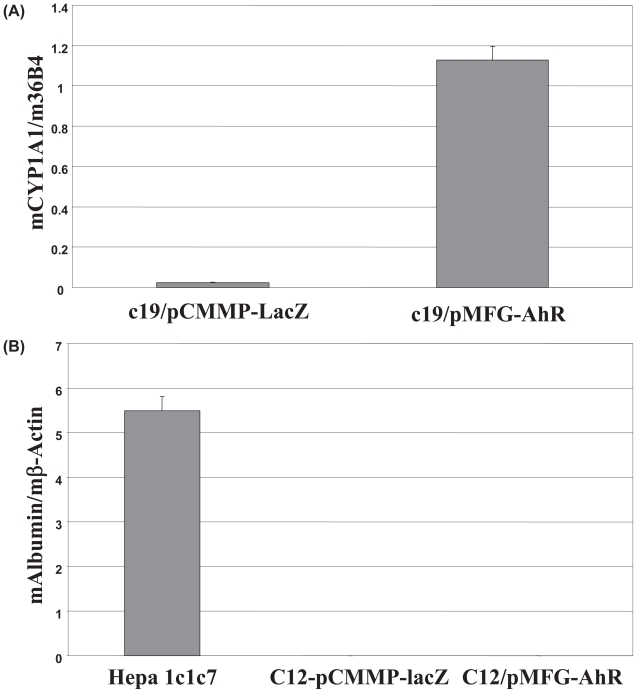
Total RNA was isolated from the indicated cells and used for reverse transcription. The cDNAs were then subjected to real time PCR analysis. The result is an average from three real time PCR reactions with the same template. Standard deviations are shown. A, Expression of Cyp1a1. The mRNA levels of Cyp1a1 were normalized to that of the constitutively expressed 36B4 gene, encoding a ribosomal subunit. B, Detection of albumin mRNA. The mRNA levels of albumin were normalized to that of the constitutively expressed β-actin gene. Standard deviations are shown.

The B mutants were isolated in our laboratory by selecting for rare clones of Hepa lclc7 cells that survived the toxic effects of benzo(a)pyrene (Hankinson, 1979; Hankinson, 1983). They are deficient in the expression of AhR (Zhang et al. 1996). To search for the genes whose expression are mediated by AhR, we re-introduced AhR into c19 cells, one of the B mutants, using the retroviral vector, pMFG-AhR, containing mouse AhR (Ma et al. 1996). Infection efficiencies were tested by X-gal staining for expression of β-gal encoded by the lacZ gene in the control retroviral vector pCMMP-lacZ (Klein et al. 2000), and reverse selection for expression of AhR contained in pMFG-AhR (Zhang et al. 1996). (The pCMMP retroviral vector is derived from pMFG and the two vectors are very similar). Both types of retrovirus infected the B mutants with nearly 100% efficiency (data not shown). We confirmed the expression of the AhR protein in c19 infected with pMFG-AhR by Western blot analysis (Fig. 1). Of the total of 12,488 genes on the microarray chip, 5491 genes were expressed in the B mutant cells. With DChip analysis, we identified 30 genes differentially expressed by at least 2-fold in c19 cells infected with pCMMP-lacZ compared with those infected with pMFG-AhR (Table 1). 22 genes were up-regulated and 8 genes were down-regulated by re-introduced AhR. These genes were clustered into stimulus response, metabolism, cell communication, and morphogenesis groups, based on the functions of their protein products. Interestingly, no genes in the cell cycle or differentiation clusters were affected by AhR expression. Cyp1a1, phospholipase A2, NAD(P)H dehydrogenase quinone 1 (NADPH quinone oxidoreductase 1), which are known to be regulated by TCDD treatment (Sutter and Greenlee, 1992; Hankinson, 1995; Boverhof et al. 2006) but are poorly inducible by dioxin in B mutant cells comparing with wild-type Hepa 1c1c7 cells (our unpublished observations), were up-regulated by AhR. We confirmed the data for Cyp1a1 from the cDNA microarray using real time PCR. As shown in Figure 2A, Cyp1a1 was indeed up-regulated significantly by expressing AhR in c19 cells. Although DChip analysis did not show any change in expression of AhR, this is explained by the fact that pMFG-AhR expresses a mRNA for the coding region of AhR, and all the 16 probes for AhR in the chip are designed to anneal to the 3′-UTR of the mRNA of AhR.

### AhR did not up-regulate expression of albumin

The parental Hepa1c1c7 cells express albumin mRNA. However, we found that the mRNA level for albumin was too low to be detected even in c19 cells forced to express AhR, as shown by DChip analysis (data not shown). This was surprising since the expression of albumin was previously reported by Ma and Whitlock (Ma and Whitlock, 1996) to be rescued by expressing exogenous AhR in an AhR-deficient clone of Hepa1c1c7 cells equivalent to our B mutants. To investigate whether this findings is cell-specific, we infected c12, another B mutants cloned from Hepa1c1c7 cells (Hankinson, 1983), with pMFG-AhR and pCMMP-lacZ, respectively. We confirmed the expression of AhR in c12 cells infected with pMFG-AhR using Western blot analysis (Fig. 1). We quantified mRNA of albumin in these cells and in the wild type Hepa1c1c7 cells using real time PCR and found that although the mRNA for albumin is readily detectable in Hepa1c1c7 cells, it was not detectable in c12 cells infected with either pCMMP-lacZ or pMFG-AhR (Fig. 2B), consistent with our cDNA microarray data.

### Re-introduction of AhR into B mutants failed to change their morphology

**Figure 3:**
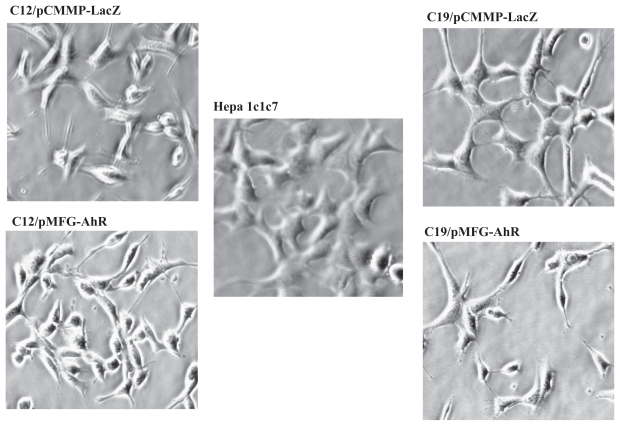
Morphology of wild-type, B mutants, and B mutants infected with pMFG-AhR and pCMMP-lacZ. The cells were cultured under normal conditions, visualized by phase contrast microscopy, and photographed

The wild-type Hepa 1c1c7 cells express a partially differentiated liver phenotype. Phase-contrast microscopy reveals that Hepa1c1c7 cells are epithelioid and polygonal, contain multiple nucleoli, have a granular cytoplasm, and form an orderly monolayer at confluence; these features are typical of hepatocytes. B mutants show morphological changes; for example, they appear less well differentiated and more spindle shaped, and they fail to form well-organized monolayers at high density (Fig. 3). These observations are similar to those made by Ma and Whitlock for their AhR-deficient derivatives. Ma and Whitlock reported that re-introduction of AhR into AhR-D cells reverted their morphology to that of Hepa1c1c7 cells. However, we did not observe any morphological changes in either c19 or c12 cells forced to express exogenous AhR.

### AhR showed little effects on the cell cycle of B mutants

To investigate if AhR affects the cell cycle of Hepa 1c1c7 cells, we performed cell cycle analysis using c19 cells expressing ectopic AhR or lacZ. We used flow cytometry to determine whether AhR exerts an effect at a particular phase of the cell cycle. However, we did not find any significant difference in the duration of any phase of the cell cycle, comparing B mutants expressing AhR and those expressing lacZ, after analysis of unsynchronized populations during logarithmic growth (Table 2). We obtained similar results with cell cycle analysis of c12 cells expressing AhR or lacZ (data not shown). These results are consistent with the data from our DNA microarray analysis that failed to show any cell cycle-related genes regulated by AhR, implying that AhR has little effect on the cell cycle of the mouse hepatoma cells in the absence of TCDD.

## Discussion

Recent studies using DNA microarray techniques suggested that approximately 300 genes were altered by TCDD-dependent AhR activation in the human hepatoma HepG2 cells (Puga et al. 2000b; Frueh et al. 2001). However, target genes for AhR in the absence of exogenous ligands remain unknown. Since AhR has been reported to modulate the phenotype of mouse hepatoma cells in the absence of exogenous ligands (Ma and Whitlock, 1996), it was of interest to further elucidate the potential mechanisms by which AhR exerts its effects on the phenotype of cells, and to identify AhR target genes in the absence of exogenous ligands. In this study, we re-introduced AhR into c19 cells, a B mutant deficient for AhR, and compared the gene expression profile of these cells with that of c19 cells with introduced lacZ. We identified 30 genes that are differentially expressed at least 2-fold in the two cell types. These genes are clustered into stimulus response, metabolism, cell communication, and morphogenesis, based on the biological functions of their protein products. Interestingly, Cyp1a1, phospholipase A2, and NAD(P)H quinone oxidoreductase 1, which were previously shown to be regulated by TCDD treatment, were up-regulated by AhR. We speculate that this result may be ascribed to “leaking through” of AhR into the nucleus, or activation of AhR by endogenous ligands. We previously provided evidence that the AhR is partially activated by ligands present in the medium in Hepa-1 cells cultured in the absence of added ligand (Wang et al. 2004). To our knowledge, the other genes we identified have not been reported to be induced by TCDD (Jin et al. 2004, Boverhof et al. 2005, Boverhof et al. 2006, Dere E et al. 2006, Yoon et al. 2006). They therefore may represent novel target genes of unliganded AhR. Among these genes, six are involved in cell stimulus response, suggesting a role of AhR in such responses. Considering that suppression of the immune response by TCDD is largely mediated by AhR, it will be of interest to ascertain the potential relationship between the AhR-induced genes related to stimulus response and AhR- mediated immune repression. Since AhR is located in the cytoplasm in the absence of exogenous ligands, it is intriguing how genes can be induced by the un-liganded AhR. One possibility is that AhR transducts its signal into the nucleus via a factor (s) that translocates into the nucleus. Furthermore, our data suggest that, in the absence of sufficient amounts of exogenous ligand, AhR can alter only a very limited number of genes and only to a very limited degree.

We further investigated whether AhR could change the phenotype of the cells. Introduction of AhR failed to alter the expression of albumin, cell morphology, or cell cycle parameters in our B mutant clones. This conflicts with the findings made by Ma and Whitlock (Ma and Whitlock, 1996). In their studies, they found that AhR restored the expression of albumin in AhR-D cells (equivalent to our B mutants) to the same level as that in wild type Hepa 1c1c7 cells, restored the morphology of AhR-D cells to the differentiated phenotype of the parental Hepa 1c1c7 cells, and reduced the length of the G1 phase of the cell cycle of the cells. It is possible that these discrepancies results from the fact that Ma and Whitlock transfected their AhR-D cells with an AhR-expressing plasmid and picked a single colony for analysis. In contrast, we infected B mutant cells with a retroviral vector expressing AhR and used a pool of infected cells to perform our studies. Cells from a single colony may not be representative of the whole cell population, since certain cellular phenotypes can exhibit considerable clonal variation in culture. It is conceivable that the AhR-transfected clone studied by Ma and Whitlock coincidentally lost albumin expression and changed morphology via mechanisms unrelated to AhR. Secondly, the AhR-D cells and the B mutants were both isolated from wild type Hepa 1c1c7 cells, but each represents an independent clone. Therefore, we cannot exclude the possibility that the effects of AhR on cell phenotype are dependent on the properties of each individual clone. This possibility receives some support from the findings that AhR exerts opposite effects on the cell cycle and the state of differentiation in different type of cells (Gudas and Hankinson, 1987; Puga et al. 2000a; Shimba et al. 2002; Huang and Elferink, 2005;). Nevertheless, in this study, we conclude that AhR, in the absence of sufficient amounts of exogenous ligands, has little effect on the proliferation or state of differentiation of mouse hepatoma cells.

## Figures and Tables

**Table 1:** List of genes that respond to AhR.

Category	Gene	Accession	fold change^a^
Stimulus Response	defender against cell death 1	AV007196	−16.44
	killer cell lectin-like receptor, subfamily A, member 1	M25775	2.03
	killer cell lectin-like receptor, subfamily A, member 4	U10090	2.23
	secretory leukocyte protease inhibitor	AF002719	−2.19
	chemokine (C-C motif) ligand 12	U50712	−2.29
	chemokine (C-X-C motif) ligand 1	J04596	−2.03
Metabolism	cytochrome c oxidase, subunit IVa	M37831	−2.11
	cytochrome P450, 1a1, aromatic compound inducible	K02588	15.78
	mannosidase 1, alpha	AI021125	2.21
	phospholipase A2, group IVA (cytosolic, calcium-dependent)	M72394	2.17
	vanin 1	AJ132098	−2.75
	NAD(P)H dehydrogenase, quinone 1	U12961	2.2
	heparan sulfate (glucosamine) 3-O-sulfotransferase 1	AF019385	2.05
	chromobox homolog 1 (Drosophila HP1 beta)	X56690	2.38
Cell Communication	gap junction membrane channel protein alpha 1	M63801	2.19
	tachykinin receptor 1	X62934	19.74
Morphogenesis	small proline-rich protein 2A	AJ005559	−2.65
Other	expressed sequence C79026	AI561567	2.18
	high mobility group AT-hook 1	AV311953	2.34
	zinc finger protein 62	Z67747	2.02
	T-cell receptor beta, joining region	M20878	4.14
	ELL-related RNA polymerase II, elongation factor	AI197161	2.6
	ESTs	AI853048	2.23
	ESTs, Weakly similar to down-regulated by Ctnnb1,	AI891514	2.59
	expressed sequence R74626	R74626	2.59
	RIKEN cDNA 2610007K22 gene	AV291989	2.71
	RIKEN cDNA 2900024N03 gene	AI508500	2.42
	RIKEN cDNA 5430432P15 gene	AI265115	2.12
	RIKEN cDNA D730042P09 gene	AV150572	−2.14
	DNA segment, Chr 3, Wayne State University 167, expressed	AA408385	5.41

The cDNA microarray data were analyzed to dertermine the number of genes that were up- and down-regulated by AhR.

a Positive fold induction represents the ratio of the signal obtained from c19 cells expressing ecotopic AhR divided by the signal obtained from the cells expressing lacZ. Negative fold induction ratios represent the signal obtained from c19 cells expressing lacZ divided by the signal obtained from the cells expressing ecotopic AhR.

**Table 2:** Cell cycle analysis^a^.

Cell type	Percentage of cells ± SD		
	G1 (%):	G2 (%):	S (%):
C19/pCMMP-lacZ	50.2 ± 5.8	10.5 ± 0.7	39.3 ± 5.5
C19/pMFG-AhR	47.0 ± 2.9	11.0 ± 1.8	42.0 ± 1.2

a Cells in mid-log phase were stained with propidium iodide, and the percentage of cells in each phase was determined by flow cytometry. The means and standard deviations were determined from three experiments.
